# Predicting Underestimation of Invasive Cancer in Patients with Core-Needle-Biopsy-Diagnosed Ductal Carcinoma In Situ Using Deep Learning Algorithms

**DOI:** 10.3390/tomography9010001

**Published:** 2022-12-20

**Authors:** Luu-Ngoc Do, Hyo-Jae Lee, Chaeyeong Im, Jae Hyeok Park, Hyo Soon Lim, Ilwoo Park

**Affiliations:** 1 Department of Radiology, Chonnam National University, 42 Jebong-ro, Dong-gu, Gwangju 61469, Republic of Korea; 2 Department of Radiology, Chonnam National University Hospital, 42 Jebong-ro, Dong-gu, Gwangju 61469, Republic of Korea; 3 Department of Medicine, Chonnam National University, Gwangju 61469, Republic of Korea; 4 Department of Radiology, Chonnam National University Hwasun Hospital, Gwangju 58128, Republic of Korea; 5 Department of Artificial Intelligence Convergence, Chonnam National University, Gwangju 61186, Republic of Korea; 6 Department of Data Science, Chonnam National University, Gwangju 61186, Republic of Korea

**Keywords:** ductal carcinoma in situ, underestimation of invasive cancer, deep learning, magnetic resonance imaging, machine learning

## Abstract

The prediction of an occult invasive component in ductal carcinoma in situ (DCIS) before surgery is of clinical importance because the treatment strategies are different between pure DCIS without invasive component and upgraded DCIS. We demonstrated the potential of using deep learning models for differentiating between upgraded versus pure DCIS in DCIS diagnosed by core-needle biopsy. Preoperative axial dynamic contrast-enhanced magnetic resonance imaging (MRI) data from 352 lesions were used to train, validate, and test three different types of deep learning models. The highest performance was achieved by Recurrent Residual Convolutional Neural Network using Regions of Interest (ROIs) with an accuracy of 75.0% and area under the receiver operating characteristic curve (AUC) of 0.796. Our results suggest that the deep learning approach may provide an assisting tool to predict the histologic upgrade of DCIS and provide personalized treatment strategies to patients with underestimated invasive disease.

## 1. Introduction

Ductal carcinoma in situ (DCIS) is a noninvasive breast cancer with the presence of abnormal cells inside a milk duct [1]. Unlike invasive ductal carcinoma (IDC) that spreads into surrounding breast tissue, the proliferation of malignant cells in DCIS is confined within the basement membrane of milk ducts [2]. Therefore, it permits relatively less-invasive therapy options compared to IDC, which usually requires axillary interventions. While core-needle biopsy (CNB) is a gold standard for the diagnosis of breast lesions, a presurgical diagnosis of DCIS using CNB with a small caliper poses a potential sampling error and may result in the upgrading of DCIS to invasive disease in the histopathology of surgically excised specimens. The percentage of DCIS at CNB to upgraded DCIS after surgery has been shown to be 6–41% [3,4].

The differentiation of pure DCIS from upgraded DCIS with invasive component is of clinical importance because the treatment strategies and prognosis of these two conditions are markedly different. Sentinel lymph node biopsy (SLNB) is not recommended for DCIS when breast-conserving surgery is planned because of the low incidence of axillary involvement in pure DCIS (1–2%) [5]. In cases of upgraded DCIS, however, SLNB or axillary lymph node dissection (ALND) is necessary. Presurgical prediction of DCIS with occult invasive component would equip clinicians with an important assisting tool to provide optimal medical care to these patients.

A number of efforts have been made to evaluate the preoperative factors that are predictive of occult invasive component in DCIS using various breast imaging modalities [6,7,8,9]. Several studies have shown that magnetic resonance imaging (MRI) has the potential to distinguish DCIS with occult invasive component from pure DCIS [6,10,11]. These studies examined the conventional MR imaging features, such as cancer size and lesion signal intensity, for identifying predictors of occult invasive component in DCIS. Recently, deep-learning-based methods have emerged as one of the most powerful tools for computerized pattern recognition in the analysis of medical images. Zhu et al. demonstrated that a convolutional-neural-network (CNN)-based algorithm using breast MRI can predict DCIS with occult invasion with a borderline performance of AUC (0.68–0.7) [12].

The aim of this study was to develop deep learning models based on breast MRI to distinguish between pure vs. upgraded DCIS diagnosed by CNB. A total of three CNN-based models, which differed in the type of input images and the CNN architectures, were developed to investigate the feasibility of using them to predict the upgrading status of DCIS.

## 2. Materials and Methods

### 2.1. Subjects

**Figure 1:**
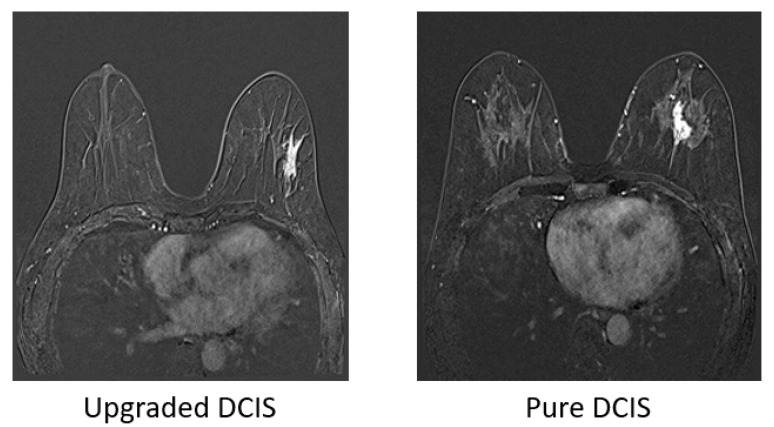
Examples of the first subtraction images (the 1st post-contrast minus pre-contrast images) for upgraded ductal carcinoma in situ (DCIS) and pure DCIS, both showing segmental heterogeneous non-mass enhancement which was hard to discriminate from each other.

This retrospective study was approved by the institutional review board (IRB No. CNUHH-2022-110), and informed consent was waived. A total of 352 lesions diagnosed with DCIS by CNB in patients who underwent preoperative breast MRI examination from 2011 to 2017 were included. We assigned the study patients into two groups: pure DCIS (*n* = 202) and upgraded DCIS (*n* = 150). When the histopathologic analysis of the surgical specimen showed microinvasive or invasive foci within a tumor, the tumor was histologically defined as upgraded DCIS. Preoperative axial T1-weighted dynamic contrast-enhanced MRI was acquired using clinical 3T scanners (Tim trio, Skyra, or Skyra II, Siemens Healthcare, Erlangen, Germany) and consisted of one pre-contrast and five post-contrast series. We used the subtraction images which were generated by subtracting pre-contrast imaging sequence from each of the five post-contrast series. The data were selected based on the assumption that only one side of the breast contains DCIS. Figure 1 exhibited examples of subtraction images from pure DCIS and upgraded DCIS. The entire datasets were divided into training (*n* = 220), validation (*n* = 60), and testing (*n* = 72) sets. Table 1 described the distribution of each set in pure and upgraded DCIS.

### 2.2. Pre-Processing

Standard normalization was applied to the original subtraction images. Based on the histogram of the 3-dimensional MRI data, the outlier removal algorithm was applied by changing the intensities of voxels with 1% upper and lower bounds to those of 1% and 99% voxels, respectively.

### 2.3. Data Preparation

**Figure 2:**
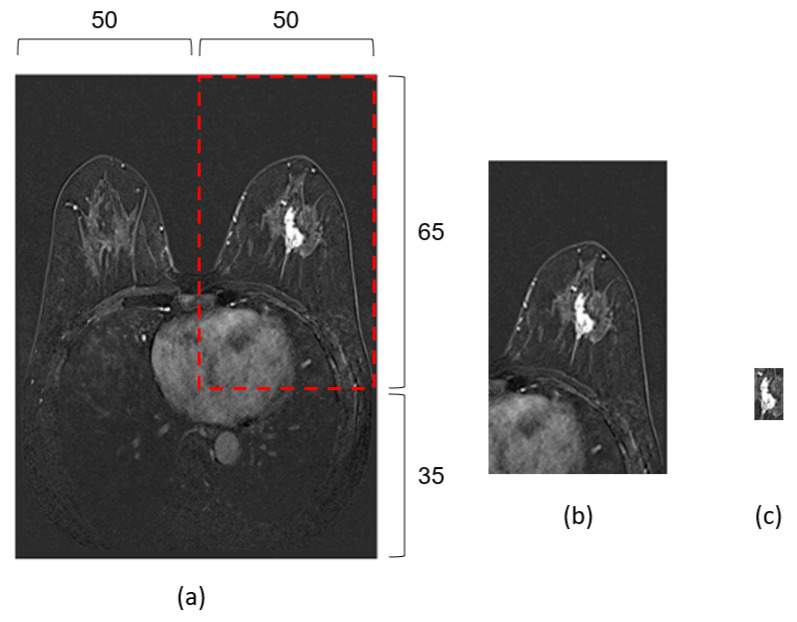
Examples of two types of data prepared for model training. The illustration of the cropping ratio (**a**). The first data is a quarter of image that contains the side of breast with tumor (**b**). The second data is an image cropped with the smallest tumor bounding box (**c**).

Two types of input data were prepared (Figure 2). Each type of data was constructed as 3-channel arrays with each channel corresponding to the 1st, 2nd, and 3rd subtraction images, respectively. First, the original subtraction images were cropped into four parts with the ratio of 65:35 in vertical axis and 50:50 in horizontal axis. The top-left and top-right quarters corresponded to two sides of breast. The quarter image that contained DCIS was selected as the first type of input data, as shown in Figure 2b. The selected quarter image could contain not only the breast with lesion, but also the background and small part of other tissues such as lung and heart. The models that were trained with the first input data were named a Detection-Transfer Recurrent Residual Convolutional Neural Network (D-T RRCNN) model because the model was first trained with a detection network for the purpose of focusing on lesion area and then fine-tuned with RRCNN. In D-T RRCNN model, there was no user involvement in defining the extent of tumor. The second type of input data tried to capture tumor mass with a minimum involvement of other tissues. Intratumor Regions of Interest (ROIs) were manually drawn on the 1st subtraction images by a breast radiologist with 4 years of experience using a 3D-slicer (http://www.slicer.org) (accessed on 1 May 2022). A 5 mm peritumor ROI was automatically created by extending the boundary of the intratumor ROI using a built-in segmentation tool in 3D Slicer, and the final tumor ROI was obtained by merging intratumor- and donut-like peritumor ROIs. The second input data were then created by cropping the images with the smallest bounding box that encompassed the three-dimensional volume of tumor ROIs. The cropping box were applied to the 2nd and 3rd subtraction images as well. The models that were trained with the second type of input data were named an ROIs model.

### 2.4. Model Development

Three types of deep learning models were constructed. The first one was the D-T RRCNN model, which was a sequential model where 3D MRI slices were considered as a sequence of 2D images. D-T RRCNN used the first type of input data: one quarter image as explained the previous section. A total of 20 imaging slices were selected from each patient based on the location of tumor. The second one was the RRCNN with ROIs model, which utilized a sequence of the second type of input data: image cropped with the smallest tumor bounding box. Similar to the D-T RRCNN model, 20 imaging slices were selected as input. The third one was the CNN with ROIs model, which was a 2D CNN model where a single imaging slice from the second type of input data (image cropped with the smallest tumor bounding box) was used as input. During the model training, the label (pure or upgraded DCIS) for a particular patient was used as labels for all input imaging slices. For the final model testing, the classification decision of a patient was made using the imaging slice with the highest output score.

The convolution blocks of the proposed models are similar to the structure of RRCNN [13], which was adapted from VGG16 [14] and ResNet [15]. The first block had two convolution layers with a kernel size of 7 × 7. The other blocks had three convolution layers with a kernel size of 3 × 3. The numbers of feature map in each convolution block were 64, 128, 256, 512, and 512, respectively. For the D-T RRCNN and RRCNN with ROIs models, the long short-term memory (LSTM) layer, which contained 512 hidden nodes, was attached to the final convolution block to capture sequential information. Before the model training, the input images were augmented by a random rotation from 10- to 30-degree, a horizontal flip, a vertical flip, and the addition of Gaussian noise. Our models were trained using TensorFlow-GPU with two NVIDIA GTX 1080 Ti. Table 2 shows the setup parameters of each model, including batch size, learning rate, input size, and the physical size of ROI bounding boxes. We used the Adam optimizer and set the number of epochs to 1000 for all models. The following sections contain the detailed description of three models.

#### 2.4.1. D-T RRCNN Model

**Figure 3:**
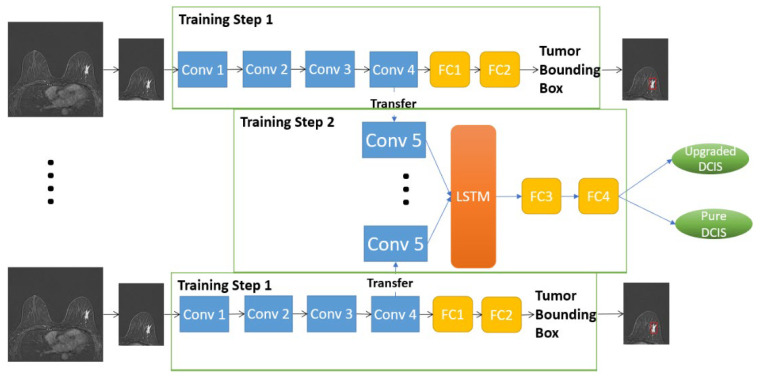
The diagram of Detection-Transfer Recurrent Residual Convolutional Neural Network (D-T RRCNN) model. The output of the Training Step 1 is Tumor Bounding Box marked by the red square. Please refer to Figure 5 for the architecture of the convolution blocks. Conv, convolution block; FC, fully connected layer; LSTM, long short-term memory.

A 2-step algorithm was implemented to apply a transfer learning method in training the model parameters. The detailed diagram of the proposed algorithm is illustrated in Figure 3. First, we designed a CNN, which received a single imaging slice as an input and performed the task of localizing the tumor bounding box containing. This network consisted of 4 convolution blocks and a regression layer was attached at the end to generate the coordinates (x, y, width, height) of the tumor bounding box (Training Step 1 in Figure 3). Second, we fine-tuned this CNN in the first step by adding an extra convolution block, followed by a LSTM layer, and replacing the regression layer with a SoftMax classifier (Training Step 2 in Figure 3). The parameters of the first four convolution blocks in the training step 1 were trained and update first. The parameters in the other layers were then updated in the training step 2. The LSTM layer was utilized for capturing sequential information through multiple MRI slices. The CNN in the first step was trained to perform the detection task and at the same time, served as a pre-training model for the classification network in the second step.

#### 2.4.2. RRCNN with ROIs Model

**Figure 4:**
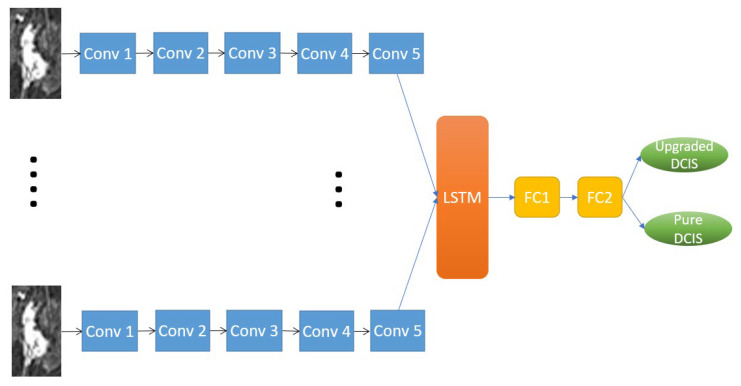
The diagram of the Recurrent Residual Convolutional Neural Network (RRCNN) with ROIs model. Please refer to Figure 5 for the architecture of the convolution blocks. Conv, convolution block; LSTM, long short-term memory; FC, fully connected layer.

Since the extent of tumor ROIs varied across different imaging slices in one patient’s MRI data, the sizes of tumor bounding boxes were synchronized throughout the entire imaging slices by expanding tumor ROIs to the size of the biggest ROI. The CNN structure used in this model is similar to the RRCNN, which contains five blocks of convolution and one LSTM layer. The diagram of the RRCNN with ROIs model is shown in Figure 4.

#### 2.4.3. CNN with ROIs Model

**Figure 5:**
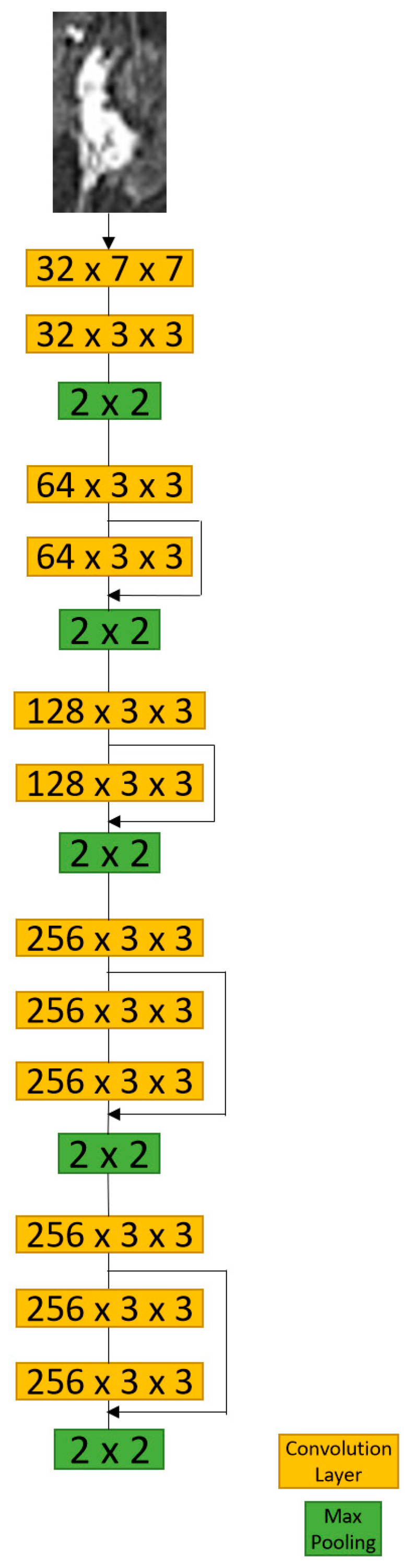
Diagram of Convolutional Neural Network (CNN) with ROIs model. Note that the architectures of the convolution blocks in Recurrent Residual Convolutional Neural Network (RRCNN) are similar to those in this model.

The input of this model was a single imaging slice with the smallest tumor bounding box. Similar to other models, the input contained 3 channels corresponded to the 1st, 2nd, and 3rd subtraction image, respectively. This model had a minor modification to RRCNN, by removing the LSTM layer (Figure 5). The imaging slices that contained DCIS with an area greater than 5 × 5 pixels were considered as input.

### 2.5. Performance Evaluation

The performance of the three deep learning models was evaluated and compared using the validation and testing datasets. Sensitivity (upgraded DCIS considered as a positive condition), specificity, accuracy with a threshold of 0.5, and the area under the receiver operating characteristic curve (AUC) were calculated.

## 3. Results

Table 3 shows the comparison of performance between the three classifiers: D-T RRCNN, RRCNN with ROIs, and CNN with ROIs models. For the validation data, the three models demonstrated comparable performances with the sensitivity, specificity, accuracy, and AUC ranging from 0.600 to 0.640, from 0.800 to 0.828, from 73.3% to 75.0%, and from 0.767 to 0.785, respectively.

**Figure 6:**
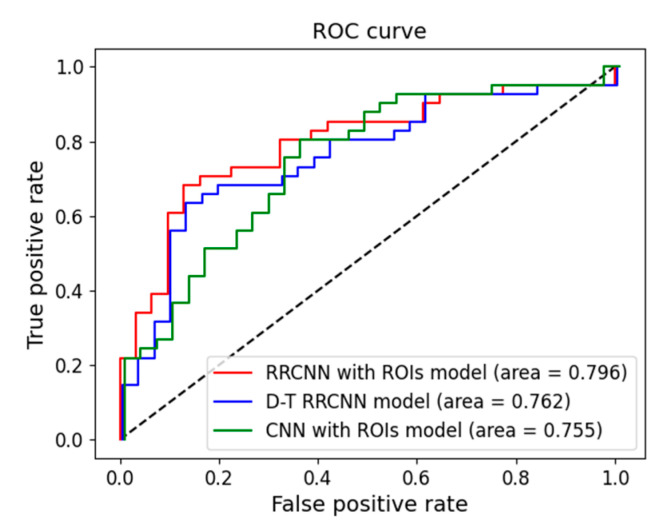
Receiver operating characteristic curves for the proposed three models: Recurrent Residual Convolutional Neural Network (RRCNN) with Regions of Interest (ROIs), Detection-Transfer (D-T) RRCNN, and 2D Convolutional Neural Network (CNN) with ROIs models.

For the testing data, the RRCNN with ROIs model achieved the highest performance with the sensitivity, specificity, accuracy, and AUC of 0.677, 0.804, 75.0%, and 0.796, respectively. The D-T RRCNN model demonstrated a performance similar to that of the RRCNN with ROIs model with the sensitivity, specificity, accuracy, and AUC of 0.645, 0.804, 73.6%, and 0.762, respectively. The CNN with ROIs model exhibited a slightly lower performance compared to those of other two models with the sensitivity, specificity, accuracy, and AUC of 0.645, 0.756, 70.8%, and 0.755, respectively. Figure 6 shows the comparison of receiver operating characteristic (ROC) curves between the three deep learning models.

## 4. Discussion

This study demonstrated the feasibility of deep learning models based on breast MRI for distinguishing pure and upgraded DCIS. Our proposed models provided the highest accuracy and AUC of 75.0% and 0.796, respectively. These results suggest that a deep-learning-based approach has a potential to be used for the accurate prediction of upgrading the status of DCIS using presurgical MRI data.

Although the differentiation of upgraded DCIS from pure DCIS is of clinical importance due to distinct treatment strategies between the two diseases, the pre-surgical prediction of DCIS with occult invasion using medical imaging data is challenging. Several previous researchers have attempted to use various breast imaging modalities, including mammography and MRI, to evaluate the preoperative factors that are predictive of upgrading DCIS [7,8,9,10,11,16,17,18]. Most of these efforts, however, have used conventional ways to analyze medical imaging data, which are based on the qualitative assessment of imaging parameters. For example, Lamb et al. reported that larger size on MRI and the presence of comedonecrosis at biopsy were significantly associated with the upgrade of DCIS [17].

Recently, a few reports have applied deep learning methods for the prediction of the upgrading of DCIS [12,19,20]. Using mammograms for the prediction of upgrading the status of DCIS, Shi et al. showed that the deep learning features from a CNN that was pretrained on non-medical images and the hand-crafted computer vision (CV) features provided comparable performances with the borderline AUCs of 0.70 and 0.68 for the deep and handcrafted CV features, respectively [20]. In another study, Hou et al. applied a deep learning model using the domain adaptation approach for distinguishing DCIS with atypical ductal hyperplasia (ADH) and DCIS with invasive component and showed a performance with an AUC of 0.697 [19]. Although MRI is the most sensitive tool for malignancy detection among different breast imaging tools [21,22], the application of deep learning approach to MRI for predicting the upgraded status of DCIS is very limited. Compared to mammogram, the larger amount of data contained in the three-dimensional format in MRI requires elaborate efforts when MRI data are used for deep learning applications. One previous study demonstrated the application of a pre-trained deep learning algorithm using breast MRI for the prediction of DCIS with occult invasion and reported an AUC of 0.68–0.70. In comparison, the deep learning models proposed in our study displayed relatively higher performances with the AUCs ranging from 0.755 to 0.796.

In this study, we tried to compare the performance between models using multiple MRI slices (sequential model, RRCNN) and a single MRI slice (2D model, CNN) as input. The performance of the RRCNN with ROIs model (accuracy = 75.0%, AUC = 0.796) was higher than those of the CNN with ROIs model (accuracy = 70.8%, AUC = 0.755). The information that was extracted between multiple MRI slices in the sequential model may have helped to assist the classification of upgraded versus pure DCIS. The high value of specificity (>0.8) from sequential models indicated that the relative information between slices plays an important role for indicating pure DCIS cases. In addition, the RRCNN with ROIs model showed a comparable, but slightly higher performance than the D-T RRCNN model (accuracy = 73.6%, AUC = 0.762). One advantage of the D-T model over the RRCNN with ROIs model is that it does require a manual annotation of ROIs. The information transferred from the detection network directly feeds the quarter of the subtraction images in the D-T model, while the ROIs models (both the RRCNN with ROIs and CNN with ROIs models) require the manual input of the lesion location. As the manual lesion annotation usually takes a great deal of human effort and time, the D-T RRCNN model may be beneficial by minimizing human involvement during the process of model training, especially when handling a large amount of data.

All three models showed relatively high specificities (0.756–0.804 on testing data), but low sensitivities (0.645–0.677 on testing data). A high level of specificity from these models means that a considerable number of upgraded DCIS were underestimated as pure DCIS. The relatively small number of upgraded DCIS (*n* = 150) compared to pure DCIS patients (*n* = 202) may have caused this imbalance between the sensitivity and specificity of model performances. In addition, the variations of pattern in upgraded DCIS and the consequent high level of difficulty in identifying it may demand more upgraded DCIS patient data for training deep learning models.

There are several limitations in our study. First, the proposed study was a retrospective effort without external test data. Future studies with an external validation from multi-center data will enhance the validity of our approach. The compilation of multi-center patient data from our collaborating institutions are currently ongoing. Second, we utilized only subtraction images to train our models. Because DCIS usually appears as non-mass enhancement, it is challenging to define tumor boundary from background parenchymal tissue in T2-weighted MRI. Further studies are necessary to properly define and delineate the lesions in T2-weighted images, and to evaluate the effects of adding multi-parametric MRI data on the predictive performance of the proposed models. Third, the process of selecting 20 imaging slices for the sequential models still required expert involvement. The total number of imaging slices contained in each MRI data can vary and only a part of the entire imaging slices from the MRI sequence contained tumors. These factors made the imaging slice selection process challenging to automate and required human involvement in the slice selection process.

The proposed models showed the feasibility of using deep learning as an assistant tool for estimating invasiveness in DCIS diagnosed by core-needle biopsy. The performance shown by sensitivity could not reach our expectations compared to the specificity. Even though we applied augmentation to make the training more balanced, the small number of upgraded DCIS patients (*n* = 150) still made the proposed models generate bias toward pure DCIS patients. The high level of difficulty and the variant of upgraded DCIS patterns also demand more upgraded DCIS patients for training a deep learning model with less bias towards pure DCIS.

## 5. Conclusions

We developed deep learning models based on breast MRI and demonstrated the feasibility of differentiating between upgraded versus pure DCIS in DCIS diagnosed by CNB. Our results suggest that this approach has the potential to be used as an assisting tool for providing personalized treatment strategies to patients with underestimated invasive disease.

## Figures and Tables

**Table 1:** The distribution of training, validation, and testing sets in two groups.

	Training	Validation	Testing	Total
Pure DCIS	126	35	41	202
Upgraded DCIS	94	25	31	150
Total	220	60	72	352

DCIS, ductal carcinoma in situ.

**Table 2:** The summary of model parameters setup for three deep learning algorithms.

	Batch Size	Learning Rate	Input Size	Size of ROI Bounding Box *
D-T RRCNN	32	3 × 10^−6^	128 × 128 × 3 × 20	
RRCNN with ROIs	32	8 × 10^−7^	64 × 64 × 3 × 20	20 × 30 (7 × 10~50 × 55)
CNN with ROIs	128	10^−5^	64 × 64 × 3	20 × 30 (7 × 10~50 × 55)

RRCNN, Recurrent Residual Convolutional Neural Network; ROIs, Regions of Interest; D-T RRCNN, Detection-Transfer Recurrent Residual Convolutional Neural Network. * in mm, mean (min~max).

**Table 3:** Comparison of performance between the three deep learning models. The 95% confidence intervals are listed in parentheses.

Models	Validation				Testing			
	Sensitivity	Specificity	Acc. (%)	AUC	Sensitivity	Specificity	Acc. (%)	AUC
D-T RRCNN	0.600 (0.408–0.792)	0.828 (0.703–0.953)	73.3 (62.1–84.5)	0.781 (0.657–0.904)	0.645 (0.476–0.813)	0.804 (0.682–0.925)	73.6 (63.4–83.8)	0.762 (0.647–0.877)
RRCNN with ROIs	0.640 (0.451–0.828)	0.800 (0.667–0.932)	73.3 (62.1–84.5)	0.785 (0.663–0.907)	0.677 (0.512–0.842)	0.804 (0.682–0.925)	75.0 (65.0–85.0)	0.796 (0.688–0.904)
CNN with ROIs	0.640 (0.451–0.828)	0.828 (0.703–0.953)	75.0 (64.1–86.0)	0.767 (0.641–0.893)	0.645 (0.476–0.813)	0.756 (0.624–0.887)	70.8 (60.3–81.3)	0.755 (0.639–0.871)

RRCNN, Recurrent Residual Convolutional Neural Network; ROIs, Regions of Interest; D-T RRCNN, Detection-Transfer Recurrent Residual Convolutional Neural Network; Acc., Accuracy; AUC, area under the receiver operating characteristic curve.

## Data Availability

The original contributions presented in the study are included in the article. Further inquiries can be directed to the corresponding authors.
